# Connecting with the community: Perceptions of a community tour

**DOI:** 10.1017/cts.2024.588

**Published:** 2024-09-06

**Authors:** Christopher Jones, Kandice Reilly, Brian Peacock, Nancy Denizard-Thompson, Alicia Walters-Stewart, Leslie Doroski McDowell, Jessica Valente, Aylin A. Aguilar, Michael Lischke, Kimberly Montez

**Affiliations:** 1 Department of Implementation Science, Division of Public Health Sciences, Wake Forest University School of Medicine, Winston-Salem, NC, USA; 2 Northwest Area Health Education Center, Wake Forest University School of Medicine, Winston-Salem, NC, USA; 3 Department of Physician Assistant Studies, Wake Forest University School of Medicine, Winston-Salem, NC, USA; 4 Department of Internal Medicine, Wake Forest University School of Medicine, Winston-Salem, NC, USA; 5 Department of Family Medicine, Wake Forest University School of Medicine, Winston-Salem, NC, USA; 6 Department of Pediatrics, Wake Forest University School of Medicine, Winston-Salem, NC, USA; 7 Department of Social Sciences and Health Policy, Division of Public Health Sciences, Wake Forest University School of Medicine, Winston-Salem, NC, USA

**Keywords:** Curriculum, community tour, neighborhood tour, social determinants of health, health disparities

## Abstract

**Introduction:**

This study explores the transformative effects of the Community Plunge, an educational program at the Wake Forest University School of Medicine (WFUSOM), on healthcare delivery, community engagement, and trainee perspectives. It addresses the broader context of health outcomes, where clinical care only accounts for 20%, emphasizing the critical role of social determinants of health (SDOH) and individual behaviors in the remaining 80%.

**Methods:**

WFUSOM’s Community Plunge, established in 2002, involves a guided tour of the community, discussions with residents, and debriefing sessions. Qualitative interviews with 20 clinicians were conducted to extract key themes and insights.

**Results:**

The study identified several key outcomes. First, participants gained crucial insights into the community’s history, structural challenges, and prevalent SDOH, enhancing their understanding of the diverse patient populations they serve. Second, the program positively influenced clinician attitudes, fostering empathy, reducing paternalism, and promoting holistic patient care. Third, participants expressed a desire for increased community involvement and reported career trajectory changes toward advocacy and volunteerism. However, challenges such as time constraints were acknowledged.

**Conclusions:**

The study advocates for collaborative efforts to enhance the program’s impact, including proactive measures to ensure respectful engagement during community tours. It positions the Community Plunge as an innovative, scalable, and transformative strategy for experiential SDOH exposure, crucial for the evolving social consciousness of healthcare learners.

## Introduction

Clinical care, including access to and quality of care, contributes only 20% toward an individual’s health outcomes, such as longevity and quality of life. The rest relies on social, economic, and environmental factors, which are the social determinants of health (SDOH) (e.g., the conditions where people are born, live, learn, work, play, worship, and age), and individual health behaviors [1]. Hence, an educational focus on biological and medical factors alone may have a limited effect on improving an individual’s health outcomes.

Increasingly, academic medical centers (AMCs) are incorporating content relevant to SDOH and health disparities (e.g., preventable differences in health outcomes associated with social, economic, and/or environmental disadvantage) [2] into medical curricula, though there is no standardized process for doing so. For example, medical schools accredited by the Liaison Committee on Medical Education must show that curricula include instruction on cultural differences, health disparities, and the SDOH. However, there are no requirements in detailing the content, format, or metrics for measurable achievement [3]. Recognizing that many trainees are not from the area in which they train, AMCs recognize the importance of connecting trainees with the community they serve to understand the local drivers of health disparities and mitigate SDOH effects on patient health [4].

One effective educational approach involves offering experiential exposure to SDOH through community immersion tours for students and trainees. These tours serve as a scalable, replicable strategy for experiential and collaborative exposure to SDOH [5]. Furthermore, community tours facilitate pedagogic partnerships between faculty, local health officials, and community leaders, fostering collaborative learning environments [6]. Community tours also present an opportunity for tour attendees to experience unique strengths and assets that exist in each neighborhood [7] While some studies have evaluated community tours within training programs, there’s a limited qualitative assessment of trainees’ perspectives [8,9].

The objective of this study was to qualitatively characterize trainees’ perspectives regarding an educational community tour, its impact on healthcare delivery and community engagement, and to gather ideas for future enhancements.

## Methods

### Setting

The Wake Forest University School of Medicine (WFUSOM), located in Winston-Salem, North Carolina, is the academic core of Advocate Health, the second largest academic learning health system in the country. The WFUSOM is dedicated to educating approximately 2500 students, residents, and fellows, encompassing physicians, basic scientists, and allied professionals. The school offers a range of degrees, including a Doctor of Medicine (MD), Doctor of Philosophy (PhD), Doctor of Nursing Practice (DNP), Master of Medical Science (MMS), and Doctor of Medical Science (DMSc), in addition to housing five joint-degree programs.

Affiliated with WFUSOM is a tertiary-care hospital with 855 beds, which includes a children’s hospital and 5 community hospitals. The medical system comprises over 300 primary and specialty care facilities, supported by a network of more than 2700 physicians. Both the medical center and children’s hospital are designated Level I trauma centers, boasting clinical expertise across more than 100 specialties. The hospital caters to a diverse patient demographic, including individuals from rural areas, racial and ethnic minorities (43% representation), and foreign-born individuals. The poverty rate among the patient population stands at 14.3% [10].

### Community Plunge

The Community Plunge, initiated by the Northwest Area Health Education Center and the Department of Pediatrics Residency Program Leadership in 2002 from the Department of Pediatrics, has evolved into an experiential learning program encompassing a community tour, patient-centered focus groups, and a debriefing session. Originally designed for pediatric interns, the program was later expanded to include various training programs within the AMC. It aims to immerse trainees beyond institutional walls, engaging them in direct conversations with community members to gain insights into community needs, assets, and perspectives.

During the Community Plunge, trainees explore areas of historical significance, deprivation, and community strengths, gaining awareness of social determinants of health and prevalent health disparities. The program guide undergoes annual updates to ensure relevance and accuracy.

Over the past two decades, the Community Plunge has been integrated into diverse training programs, including residents in internal medicine, family medicine, pediatrics, and psychiatry, as well as physician assistant students, and the Clinical Translational Science Institute. While the timing of the Plunge varies across programs, it is typically conducted early in training, often during orientation, to establish foundational insights.

The Community Plunge comprises a 4-hour activity with three key components:

- A guided tour of Winston-Salem’s business and residential areas, emphasizing community development, population needs, and assets such as available programs, resources, and services,
- Focus groups with representatives from diverse populations, focusing on racial and ethnic diversity, socioeconomic status, and health concerns, and
- A debriefing session to reflect on the Plunge experience and focus group discussions, linking these to future clinical practice.

The Community Plunge shares common elements listed above but varies by each department (see Supplemental materials – Description of the tour iterations). For example, the Internal Medicine Residency Program conducts the Plunge with 10–12 residents and 2 faculty traveling in a shuttle van. The faculty conduct the speaking elements of the Plunge and offer their own experiences with and stories about the community. Learners are encouraged to ask questions throughout the Plunge. After completing the community van tour, the group stops at a local nonprofit community agency to meet community members for the focus group session of the Plunge. Focus group participants are identified from the community agency, with a special emphasis on participants referred to that particular community resource by the primary care physician. Faculty are provided a facilitator sample guide to foster the community member focus group conversation, such as “What are your barriers to accessing healthcare? What are the biggest challenges you see facing your community? What do you value when you visit your doctor?” After completing the Plunge, participants then debrief the entire Plunge experience with faculty and their co-participants using the following questions: “What reactions did you have after the Community Plunge? What are some lessons you learned? How will this change your practice of medicine?” The focus group lasts approximately 1 hour.

In contrast, the Pediatrics Residency Program Plunge is led by faculty traveling by bus in groups of 12–16 interns. During the bus portion of the Plunge, participants have no interaction with community members, only during focus groups. Focus group participants are recruited by local community-based nonprofits and are compensated, including a meal and $25 gift card. Focus group members include relevant community members pertaining to the program’s patient population, such as Spanish-speaking caregivers, caregivers of children with special healthcare needs, and teen parents. The session is led by a faculty facilitator, who follows a prepared question guide. Participants are encouraged to ask community members questions during the session. The focus groups last approximately 30 minutes for each population.

The Family Medicine Residency Program provides the Plunge as a driving windshield tour in small groups of interns in private vehicles led by faculty and chief residents. There is a prepared script and a discussion during the driving portion. The Plunge also features 30-minute focus groups at three different community stops during the driving windshield tour. Focus groups are led by a community site leader who provides an overview of the services and hosts a question-and-answer session that lasts 45 minutes. Each session concludes with a debrief session facilitated by faculty who attend the Plunge.

### Participants and data collection

Inclusion criteria included English-speaking medical providers who were former or are current trainees (e.g., students, residents, fellows) within the WFUSOM learning healthcare system and participated in the Community Plunge within the last 15 years within the specialties and programs listed above. The sampling strategy was purposive based on inclusion criteria, training program, time out of training, and participant consent. Participants were recruited via email and opted into the study by signing up for an interview using an online scheduling program.

Through a detailed review of the literature [8,11] and input from the institution’s professional qualitative research team, we developed an interview guide to elicit perspectives regarding the Community Plunge, including perceptions of and engagement with the community, its impact on healthcare delivery and community engagement, and ideas for future improvement. The interview guide was pilot tested for face validity and modified iteratively. Two nonphysician researchers (KR, AA) trained in qualitative interview techniques, who were not connected to the training programs and were naive to the participants, conducted telephone-based semi-structured interviews in English using the interview guide. Informed consent was obtained by telephone. Participants were interviewed between April and August 2023. Interviews lasted approximately 14 minutes (range 9–29 minutes). Interviews were audio-recorded, transcribed verbatim, verified against the audio, and de-identified. Interviews were conducted until thematic saturation was reached. Each participant was compensated with a $25 gift card, provided by email after completion of the interview.

### Data analysis

Raw narrative data from the interview transcripts were entered into Atlas.ti version 23 software (Scientific Software Development GmbH, Berlin, Germany) for data analysis. A coding scheme and dictionary were developed from the first five interviews. We used a combined inductive-deductive thematic analysis approach to code interviews, a technique that systematically describes qualitative data [12]. Codes were derived deductively from the research questions and the interview guide and were also created inductively as the code emerged from the data. Two qualitative researchers (KR, AA) coded each transcript independently and assigned codes to specific responses in each transcript based on the coding scheme. Discrepancies in coding were discussed among the coders and resolved iteratively. The codebook was adjusted, as needed, based on discussions of code meanings and applications. Segments of text were reviewed by code or groups of codes and summarized. Summaries were synthesized into themes using the principles of thematic analysis [13]. The WFUSOM Institutional Review Board approved this study.

### Findings

The sample consisted of 20 practicing clinicians, a majority of whom were physicians (*n* = 19), currently in training (*n* = 14), with less than six years of clinical experience (*n* = 18). The sample is further described in Table 1. The interviews revealed four themes: (1) The Community Plunge provided insights about the city’s history, structural contexts, and community challenges. (2) Clinician attitudes toward patient care and clinician actions were positively influenced. (3) Most participants were already supporting their community and shared desires to be more involved. (4) Participants also shared suggestions for future iterations of the tour.

**Table 1.** Sample demographics, *N* = 20

	*N*	Percent
**Clinical role**		
*MD/DO*	19	95%
*PA*	1	5%
**Current status**		
*Trainee*	14	70%
*Non-trainee*	6	30%
**Years in practice**		
*0–5*	18	90%
*6–10*	1	5%
*11–20*	1	5%
**Race**		
*Non-Hispanic White*	9	45%
*Non-Hispanic Black*	6	30%
*Asian*	2	10%
*Multiple races*	3	15%
**Gender**		
*Female*	13	65%
*Male*	7	35%
**Current clinical focus**		
*Family medicine*	3	15%
*Geriatrics*	1	5%
*Adult hematology oncology*	1	5%
*Internal medicine*	7	35%
*Pediatrics*	8	40%
**Years since most recent Community Plunge**		
*Less than 1*	10	50%
*2–5*	6	30%
*6–10*	3	15%
*11–15*	1	5%
**Number of tours**		
*Once*	15	75%
*More than once*	5	25%

MD = medical doctor; DO = doctor of osteopathy, PA = physician assistant.

### Insights about the community

Participants discussed insights gained from the Community Plunge about the overall community and neighborhoods they visited. Many participants shared that the tour was “helpful” in familiarizing them with the area since they moved to the area for residency and did not have spare time to explore the city. A few participants were already familiar with the city but still acknowledged that the Community Plunge allowed them to gain insight into their patients’ lived experiences or provided an opportunity to explore new neighborhoods. Several participants mentioned the historical context of the community, including city planning, systematic segregation, and “historical injustices” (P11) that have contributed to racial and health inequities and a still segregated city. Participants described divides in the community by race and culture, socioeconomic status, and access to resources.

Some participants considered the different populations that make up the community, often noting the heterogeneity of the population. A few participants also gained a better understanding of the specific populations that rely on safety net health clinics. Such populations included racial and ethnic minorities and “underserved” groups, “undocumented immigrants,” and “low-income patients.” Participants considered the impact of various community barriers that they learned about during the Community Plunge, including access to healthy food, healthcare barriers, safety and housing concerns, and transportation. Nearly all participants noted inequitable food access in the community. Several described parts of the community as “food deserts,” noting the sheer lack of “full” or “standard” grocery stores in certain regions. A few participants described that observing inequitable access to grocery stores challenged the “illusion” (P14) that patients have control over their health behaviors and thus, health outcomes – an illusion that neglects the environments and systems that patients live within.

Several participants identified health-related barriers that community members can face when accessing care, such as understanding health information, affording appointments and medications, or engaging in health-promoting behaviors. Some participants identified “the struggles that patients have in affording their healthcare,” (P16) highlighting medication and appointment costs and “insurance issues” (P03). A few participants described perceptions of mistrust as a barrier to care, stemming from the historic segregation of hospitals and disparate representation among healthcare providers.

Some participants also shared safety concerns within the community, pointing out safety and health risks associated with using public transit alone or with children and the lack of infrastructure and lighting for pedestrians in specific areas.

Participants also identified housing challenges, including access to affordable shelter and health concerns associated with unsafe or overcrowded housing. A few participants discussed environmental hazards, such as poor air quality and overcrowded houses, that could contribute to health outcomes like sleep and respiratory health. One participant addressed the “long waitlist” (P13) for community members who need access to affordable housing.

All participants described transportation barriers that community members face when attempting to seek care or go about their daily activities. Participants spoke about what they observed on the Community Plunge and how transportation can be better contextualized for providers.

Many participants specifically identified challenges with the existing public transit and infrastructure in the city, predominantly highlighting the limitations of the transit system (e.g., few bus stops, lack of access, unsafe waiting areas, bus schedules), difficulties securing groceries on buses, and the built environment that limits walkability (e.g., the dividing highway).

Several participants noted community strengths after participating in the Community Plunge. For example, they reported being better informed about existing community assets and support systems. Participants reported an increased awareness of referable resources that reduce access barriers. For instance, a few shared that they were able to make more specific or convenient recommendations based on knowing the area better. Participants were empowered to share food access resources including local and internal food pantries and federal aid programs like SNAP (Supplemental Nutrition Assistance Program) and WIC (Special Supplemental Nutrition Program for Women, Infants and Children). Some participants indicated ways that they help patients better access and afford healthcare, including cost assistance, financial counseling, and insurance registration aid. One participant shared how because of the Community Plunge, they were now more acutely aware of the registration processes for some commonly used resources and, therefore, could provide more specific guidance to patients about registering.

### Clinician attitudes and actions

Participants described the impact the Community Plunge had on their attitude toward patients. A few participants spoke about more general impacts on their practice, such as making them a “better provider” (P07) for being more acutely aware of their community’s challenges and experiences. One participant depicted the Community Plunge as an “anchor” (P10) that reminds clinicians why they went into medicine. P11 described the tour as an opportunity to rethink the way providers frame themselves in the community – as “partners rather than as saviors.”

Most participants highlighted specific ways that the Community Plunge impacted their attitudes toward patient care, including a heightened sense of empathy, more context around patient lives, a more holistic focus on the whole patient, and a deeper recognition of the barriers patients face in accessing care.

Several participants shared that the Community Plunge encouraged them to be more “empathetic” (P07) or “understanding” (P14) in their clinical practice. A few participants used the phrase “meeting patients where they are” to depict their attitude toward patient care (P18, P20).

Many participants described the benefit of having more insight into their patients’ lives, alluding to the environmental factors that impact patients’ options, choices, and behaviors. For instance, P04 explained that understanding the social factors and access to resources can reveal important details about a patient’s understanding of their health and illness. Another participant indicated that the Community Plunge reminded them to consider the contextual factors in a patient’s life without allowing their implicit or explicit biases to impact their care.

Some participants emphasized the need for a more holistic perspective to better care for their patients. P11 spoke about bringing “humanity” back to their patients in learning more about their lives outside of the 15-minute clinical encounters.

Participants shared ways that the Community Plunge impacted their clinical practice. They described additional steps that they take in a clinical setting to reflect what they learned about their patients. Key actions included offering more flexible or considerate scheduling and assistance with appointments, sharing resource referrals and recommendations, and directing patients to internal resources such as the clinic food pantries.

One barrier for clinicians to implement screening for SDOH in the clinic setting has been what to do with that knowledge. Following their Community Plunge experience, some participants focused more on screening for SDOH, asking their patients questions about their food access, transportation, safety, and housing. One participant expressed confidence in screening their patients because they are now more familiar with what is offered in the community and can share those resources.

A handful of participants explained that the Community Plunge did in fact impact their career trajectory. Some pointed to a heightened desire to engage in advocacy or community work. A few participants broadly expressed interest in working with, learning more about, and serving the community.

The Community Plunge also encouraged a few clinicians to consider where they want to practice, whether that be at an academic institution versus a private practice, or in primary care versus being in the hospital. One participant described the tour as providing an “eye into another way to practice medicine,” explaining that seeing the community like that made other career options “more attainable” and “tangible” (P12).

### Working in the community

Most participants shared instances where they supported their community outside of their required clinical work, including volunteering with community organizations, free or low-cost health clinics, and health fairs. Community health fairs were described as a good opportunity to practice their clinical care, show their face in efforts to gain community trust, and give back to the community (P07). Several participants volunteered with local nonprofit groups. Such experiences focused on health topics like pediatric safety and injury prevention, youth empowerment and self-esteem, harm reduction, and cancer screening promotion.

A few participants described ways that they give back within their clinical practice, such as spending additional time assisting patients with insurance, legal concerns, and food access. P02 explained that they helped to establish a hospital food pantry for patients experiencing food insecurity.

Some participants specified that their community work was something they had been doing for a long time, prior to the Community Plunge. These participants listed their volunteer activities like assisting with food and coat drives; fostering children in the area; donating medications and school supplies; donating financially to nonprofit organizations, like Crisis Control Ministry, a social services organization that helps with basic life needs to those facing a crisis in surrounding counties; and supporting racial and ethnic minority-owned businesses. For these participants, the Community Plunge did not affect the work they had already been doing.

A couple of participants explained that during their residency training, they did not have free time to give back to the community and, thus, shared that their way of giving back was by giving patients “proper care” (P12) and giving “the best care” that they can (P20). A few others expressed a similar view but offered their future intentions to be more involved with community organizations and policymaking when they were more established in their career.

### Suggestions for future iterations

The most popular suggestion was to offer the Community Plunge more than once or to continue the education through another means, such as an online course. Having the ability to engage in the experience multiple times was thought to enhance the clinician’s familiarity with the community and resources. (Note: A couple of participants were able to participate multiple times in the program by volunteering to help deliver the Community Plunge.) A few participants had no suggestions to offer, claiming that the Community Plunge met their expectations and needs. One participant suggested having a more generalized introduction to the community early in training and following up with a more in-depth tour later. Another popular suggestion was to provide the resources that were covered in a more accessible way, whether it was in a hard copy format or a link to be referenced later. Some participants expressed desires for a more interactive experience, such as engaging one-on-one with community organization leaders or residents who have used these resources. Finally, a few participants emphasized keeping the tour updated by describing how communities are changing through construction and other city updates.

## Discussion

Clinicians engaging in the Community Plunge gained new insights into the community’s dynamics, fostering a newfound appreciation for historical contexts, systemic neighborhood divisions, and community assets. Disparities in resources highlighted barriers to care, including affordable housing, cost of healthcare, transportation and food access challenges, and health literacy, as well as obstacles in individual neighborhood environments, such as public safety and environmental concerns. The Winston Weaver fertilizer plant fire in the Community Plunge-toured neighborhoods revealed the community within one mile of the fertilizer plant is in the 91st percentile nationally for exposure to fine particulate matter in the air, the 93rd percentile nationally for exposure to ozone, and the 92nd percentile for cancer risk from air quality, according to the EPA’s screening tool [14]. Conversely, the Community Plunge provides a means for directly observing each neighborhood’s unique strengths and assets like social services organizations, built infrastructure, and formal and informal community collaborations.

Community Plunge participants reported an impact on their attitudes toward patients, describing reassurance in their decision to practice medicine and increased empathy for patients’ living experiences and backgrounds. They also reported increased patient-centeredness, describing a desire to view the patient as a whole person with individual challenges and a need to meet them “where they are” without judgment. Clinicians further described a desire to modify their clinics to promote accessibility based on barriers observed during the Plunge. Other action steps mentioned included offering flexible appointments, assistance with scheduling, and providing thoughtful referrals to intraorganizational and community resources. Some participants noted impactful changes to their career trajectory, including a desire to participate in advocacy, community organizing, and volunteer activities. However, some participants described challenges such as existing commitments and a lack of available time to fully participate in community activities.

Drawing parallels with similar studies on community tours among trainees, our findings highlight the importance of community-based tours in fostering diverse perspectives through experiential learning [6,9,15]. Classroom curricula and clinical rotations alone may not be sufficient to meaningfully engage trainees in understanding and exploring the SDOH and health disparities of the patients they serve while also recognizing community strengths. Our findings revealed that trainees derived substantial benefits from informal discussions both during the driving portion of the Plunge and during focus group discussions with community members. A similar study demonstrated the positive impact of self-reflexive learning exercises on medical students during community-based guided tours of disadvantaged neighborhoods, showing that such exercises can significantly enhance the social consciousness of learners [9].

As medical training programs increasingly adopt curricula focused on SDOH and health disparities, community tours provide an innovative and easily scalable strategy for experiential exposure to SDOH, health disparities, and community assets. However, despite the benefits of community tours, caution must be exercised, given the potential for reinforcing negative stereotypes and risking the appearance of voyeurism, especially when touring low-income or predominantly racial and ethnic minority communities. For our Community Plunges, these efforts have included maintaining manageable-sized groups where a facilitator can ensure participants effectively engage with the material through discussion and reflection, including how to apply what was learned during the Plunge to future practice, as many interview participants mentioned. In addition, care is taken to highlight positive aspects of each neighborhood in contrast to detractors of health. Some Plunges begin with a primer that includes community health workers who educate participants about institutional and community partnerships designed to support patients’ psychosocial needs. Care must be taken as touring communities without meaningful engagement with members of the community may unintentionally foster narrow and discriminatory perspectives, which the focus groups help to address [16]. Collaborative efforts involving local health officials, community members, and community-based organizations can enrich the learning environment and enhance understanding of the SDOH, health disparities, and community strengths [15]. Additionally, facilitators can sufficiently prepare trainees before the tour and debrief after the tour using self-reflexive activities [17]. Involving community members in the debrief session may expose trainees to additional perspectives, although doing so may prevent trainees from speaking more freely. Future research can explore community members’ attitudes toward tour participation to prevent objectification or ethical concerns. Proactive measures ensure respectful engagement, fostering genuine understanding, empathy, and compassion [9].

### Limitations

The study was limited in a few ways. While most of the participants completed the Community Plunge within the last year, a few participants had completed the Community Plunge several years ago, which may have introduced recall bias. Although the sample was relatively diverse in clinical focus and race and ethnicity, it lacked heterogeneity in some ways, such as gender. Additionally, the sample was overly representative of physicians compared to non-physician providers. Similarly, the sample mostly consisted of those very early in their career who had participated in the Community Plunge in the last 1–3 years. However, this helped limit the impact of recall bias for most of the sample. All participants included in the study were from one institution, so our results may not be transferable to another institution. The modest sample size limited our ability to assess experiences by subgroup due to difficulties with recruitment. However, the sample was sufficient to reach thematic saturation, aligning with recommendations from a recent systematic review that found saturation is typically reached between 9 and 17 interviews [17]. Furthermore, participants who agreed to participate in an interview may not be representative of all trainees who participated in the Community Plunge. Additionally, community asset mapping has not been a component of the Community Plunge yet could provide more context to participants about available services and resources. Lastly, interview participants were not asked about their experiences with focus groups or debriefing sessions. Input about these experiences would provide additional content to this subject and should be included in future surveys.

### Challenges

Some challenges have been noted by administrators of the Community Plunge, including keeping the activity guide updated each year to incorporate changes and new observations and securing funding for transportation, food, faculty time, and focus group participant remuneration. Typically, grants and departmental funds have been utilized to support these expenses. Recruitment of focus group participants has been challenging. Additionally, identifying and securing partnerships with community-based organizations that work closely with relevant patient populations has been difficult.

### Future directions

Community tours offer valuable insights and opportunities to engage with the patients and communities that trainees serve by experientially developing an awareness of how the SDOH and health disparities must be recognized and addressed, while also acknowledging community resources. Future research on experiential learning tours should also explore the integration of virtual reality or augmented reality technologies to deliver the tour experience, offering learners immersive simulations that transcend traditional boundaries and eliminate logistical concerns of location-based tours. Additionally, a shift toward interprofessional education represents a transformative opportunity for research, envisioning tours that bring together diverse healthcare professionals, social workers, educators, and community leaders to simulate real-world collaborative environments. Immersion tours, which feature arts and cultural experiences, are another future direction that can provide a holistic understanding of communities. In response to emerging health challenges, maintaining adaptability is key, ensuring that experiential community tours address current problems, such as public health crises, emerging diseases, or evolving social and economic dynamics affecting healthcare. It has been noted that arranging logistics for the Community Plunge with key referral agencies (Crisis Control Ministries, YWCA Gateway to Success, Healthcare Access, etc.) during times of crisis, such as the COVID-19 pandemic, has served as a venue for education, fostering continued partnerships and patient referrals. Community asset mapping should be considered as an addition to the tour to provide additional context to learners embarking on the tour. Lastly, the future may see a stronger emphasis on cultivating advocacy skills among learners, empowering them to advocate for policy changes, community resources, and healthcare equity, thereby nurturing socially responsible and proactive healthcare professionals.

## Conclusion

The Community Plunge provides a replicable model for AMCs and other organizations to build empathy, instill compassion, and foster self-reflection. It is especially valuable when participants are shaping their careers and establishing their identities as community members and health professionals. Our results suggest that this community-based tour promotes self-reflection and understanding of one’s community and encourages participation in community-based solutions. The Community Plunge emerges as a powerful educational tool for addressing SDOH and health disparities. By incorporating self-reflexive exercises and considering multidisciplinary perspectives, future iterations can maximize their effectiveness. These insights are not only applicable in clinical settings but also contribute to a broader understanding of health equity.

## Supporting information

Jones et al. supplementary material Jones et al. supplementary material

## Supplementary material

The supplementary material for this article can be found at https://doi.org/10.1017/cts.2024.588.
