# Supplementary material for: Connecting with the community: Perceptions of a community tour
Source: J Clin Transl Sci. 2024 Sep 6;8(1):e113. doi: 10.1017/cts.2024.588 (PMC11428111; doi:10.1017/cts.2024.588)
Supplement: Jones et al. supplementary material [file S2059866124005880sup001.docx]

**Supplemental Table:** *Description of the various Community Plunge Iterations*

| **Department** | **Frequency** | **No. of Attendees** | **Description of the Attendees** | **Description of the Tour** |
| --- | --- | --- | --- | --- |
| **Clinical and Translational Science Institute** | Twice annually | 12 | Clinicians, researchers, and staff | - Focus on community resources - Narrative windshield tour; 4-5 community stops - Debrief as a group |
| **Family Medicine** | Annually | 13 | Interns | - Focus on community resources - Narrative windshield tour; 3 community stops - Debrief as a group |
| **Health Equity Certificate Program** | Annually | 10-12 | Interns, medical students, residents, PA students | - Focus on community resources and historical context of city - Narrative windshield tour |
| **Internal Medicine** | Annually | 12 | Interns | - Focus on community resources; - Narrative windshield tour; faculty conduct speaking elements, ask standardized discussion questions. - Debrief as a group at local non-profit community agency. |
| **Pediatrics** | Annually | 16 | Interns | - Focus on community resources and historical context of city - Narrative windshield tour - Debrief as a group - Focus groups with patient population |
| **Primary Care** | Quarterly | 12 | Interns | - Focus on community resources - Narrative windshield tour; 3 community stops - Debrief with population health community health workers, patient navigators, and social workers |
| **Physician Assistant (PA) Program** | Annually | 54 | First-year PA students | - Narrative windshield tour - Debrief as a group |
